# Beyond the Meninges: A Unique Cardiac Complication of Streptococcus pneumoniae Meningitis

**DOI:** 10.7759/cureus.53967

**Published:** 2024-02-10

**Authors:** Yazan Alamro, Khurram Arshad, Rabia Latif, Antoine Egbe Bessong Tabot, Najeebullah Bangash

**Affiliations:** 1 Internal Medicine, Corewell Health East, Dearborn, USA; 2 Internal Medicine, McLaren Flint Hospital, Flint, USA; 3 Cardiology, Corewell Health East, Dearborn, USA

**Keywords:** hiv aids, sinus bradycardia, intracranial hypertension, streptococcus pneumoniae, meningitis

## Abstract

We present a case of a 30-year-old male who was admitted with Streptococcus pneumoniae meningitis. He initially responded well to antibiotic therapy. However, the patient later developed symptomatic sinus bradycardia, which was likely due to intracranial hypertension. Even though the patient showed overt symptoms, vigilant monitoring, interdisciplinary collaboration, and cardiac evaluation helped avoid unnecessary interventions. This case highlights the importance of recognizing and managing rare cardiac complications associated with central nervous system infections.

## Introduction

Sinus bradycardia is characterized by the sinus node initiating cardiac muscular depolarization, resulting in a heart rate of less than 60 beats per minute (bpm). To diagnose this condition, an electrocardiogram (ECG) must reveal a normal sinus rhythm with a rate below 60 bpm [1]. In rare instances, sinus bradycardia may manifest as a complication in the context of central nervous system (CNS) infections. We report a case of a patient with Streptococcus pneumoniae meningitis who developed symptomatic sinus bradycardia associated with slightly increased intracranial pressure.

## Case presentation

**Figure 1:**
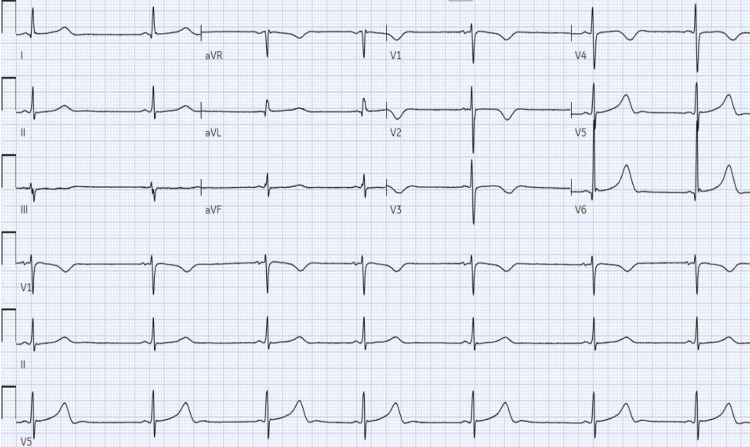
Marked sinus bradycardia with prolonged QT intervals.

**Figure 2:**
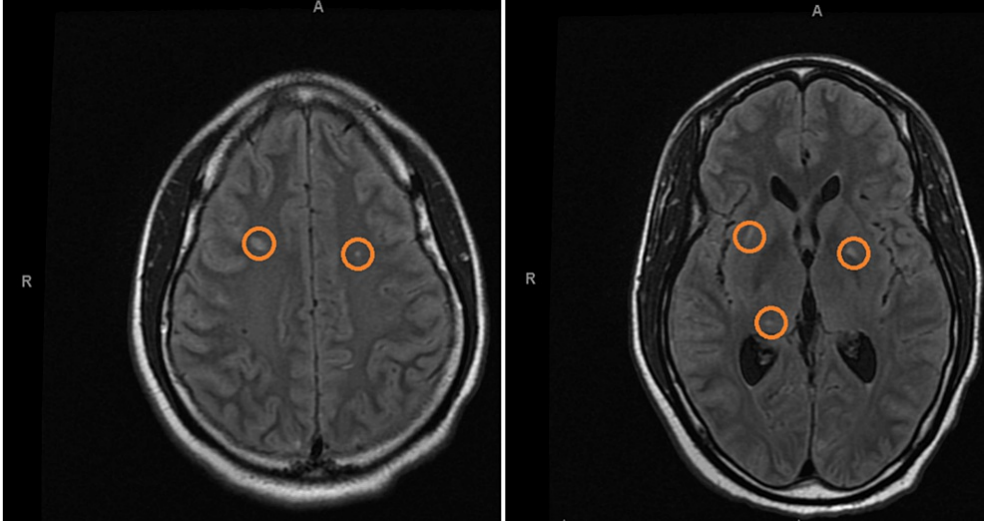
There are numerous small foci (orange circles) of the T2 hyperintense signal with restricted diffusion involving the left putamen, and corona radiata are scattered in the bilateral frontoparietal white matter. Findings are most compatible with acute/subacute ischemic infarcts and vasculopathy. No significant associated mass effect. The ventricles are normal in size.

A 30-year-old man was admitted to the hospital with fever and confusion. Upon examination, he displayed a Glasgow Coma Scale of 14 out of 15, was febrile, and had nuchal rigidity. Presumed bacterial meningitis prompted immediate treatment with ceftriaxone, vancomycin, and dexamethasone. Lumbar puncture revealed pleocytosis, low CSF glucose, and elevated protein, confirming bacterial meningitis with an opening pressure of 21 cm H_2_O and 704 white blood cells per field with 91% neutrophils. An antigen test confirmed Streptococcus pneumoniae as the causative agent. On the second day, the patient became bradycardic with the initial EKG given in Figure 1 showing sinus bradycardia (30-35 beats per minute with Qt prolongation along with dizziness and hypotension (blood pressure at 87/52 mmHg). Intracranial hypertension etiology was suspected and supported by MRI findings given in Figure 2. Fundoscopy and transthoracic heart ultrasound were unremarkable. Atropine was administered several times. Once the meningitis symptoms completely resolved, no bradycardia recurrence was observed during follow-up.

## Discussion

This case presents a rare and intriguing association between Streptococcus pneumoniae meningitis and the development of sinus bradycardia. Bacterial meningitis, a severe inflammatory condition affecting the meninges, typically presents with characteristic symptoms such as fever, altered mental status, headache, and nuchal rigidity [2]. Our patient, in line with the clinical presentation, was promptly treated with ceftriaxone, vancomycin, and dexamethasone, following the American guidelines for bacterial meningitis [3], resulting in a clinical response. The development of symptomatic sinus bradycardia, however, poses a unique clinical challenge. 

Sinus bradycardia, defined as a sinus rhythm of less than 60 bpm, can be a normal physiological response but is also associated with various pathological conditions [4]. In our case, potential causes such as ischemic heart disease, sick sinus syndrome, metabolic factors, medications, and toxins were considered [4,5]. However, a thorough investigation ruled out these common etiologies. Medications administered were unlikely contributors, and a transthoracic heart ultrasound revealed normal cardiac function, excluding structural abnormalities or pericardial effusion. 

Meningitis, an inflammation of the protective membranes around the brain and spinal cord, doesn't directly cause bradycardia (slow heart rate). However, complications from meningitis, like increased intracranial pressure, can affect the autonomic nervous system, influencing heart rate. The inflammatory response triggered by meningitis may release substances that impact cardiac function, potentially leading to bradycardia. If the infection reaches areas regulating heart rate in the central nervous system, bradycardia may occur.

Intriguingly, the leading candidate for the development of bradycardia in our case was meningitis causing intracranial hypertension, supported by a slight elevation of the opening pressure during lumbar puncture and MRI findings indicating CNS parenchymal and vascular involvement. This aligns with existing literature highlighting the role of CNS infections, including Pneumococcal meningitis, in causing direct cytotoxic damage, oxidative stress, and subsequent intracranial complications, such as increased intracranial pressure and neuronal injury [6,7]. Vasculitis and subsequent vasculopathy can be seen in up to 25% of meningitis cases [8]. However, fundoscopy did not reveal signs of increased intracranial pressure, adding complexity to the diagnostic picture. 

The transient nature of the bradycardia, its resolution without specific intervention, and the absence of recurrence during follow-up after discharge underscore the need for caution in interpreting and managing cardiac manifestations in the context of CNS infections. While intracranial hypertension remains a plausible cause, the exact mechanism linking CNS infections to sinus bradycardia warrants further investigation. This case emphasizes the need for vigilant cardiac monitoring in patients with CNS infections. Interdisciplinary collaboration is essential to avoid unnecessary interventions.

## Conclusions

This report highlights a rare complication called sinus bradycardia, which is associated with Streptococcus pneumoniae meningitis. Although the patient responded well to immediate antibiotic and steroid therapy, the improvement of bradycardia posed a diagnostic challenge. The absence of any identifiable reversible factors and the spontaneous resolution of bradycardia without specific intervention underscore the importance of cautious management. It is possible that the patient's clinical and radiological findings indicate intracranial hypertension as the cause. This case underscores the need for clinicians to be aware of potential cardiac complications following CNS infections and the importance of a multidisciplinary approach for accurate diagnosis and tailored management. Further research is necessary to understand the mechanisms underlying such cardiac manifestations in the context of CNS infections.
